# Non-Alcoholic Fatty Liver Disease and Extrahepatic Cancers: A Wolf in Sheep’s Clothing?

**DOI:** 10.3390/curroncol29070356

**Published:** 2022-06-25

**Authors:** Athanasia Mitsala, Christos Tsalikidis, Konstantinos Romanidis, Michail Pitiakoudis

**Affiliations:** Second Department of Surgery, University General Hospital of Alexandroupolis, Democritus University of Thrace Medical School, Dragana, 68100 Alexandroupolis, Greece; ctsaliki@med.duth.gr (C.T.); kromanidismed@gmail.com (K.R.); pterion_ts@yahoo.gr (M.P.)

**Keywords:** non-alcoholic fatty liver disease, colorectal adenomas, colorectal cancer, extrahepatic cancers, metabolic syndrome, insulin resistance

## Abstract

Non-alcoholic fatty liver disease (NAFLD) is now considered the main driver and leading cause of chronic liver disease globally. The umbrella term NAFLD describes a range of liver conditions closely related to insulin resistance, metabolic syndrome, diabetes mellitus, obesity, and dyslipidemia. At the same time, several malignancies, including hepatocellular carcinoma and colorectal cancer, are considered to be common causes of death among patients with NAFLD. At first, our review herein aims to investigate the role of NAFLD in developing colorectal neoplasms and adenomatous polyps based on the current literature. We will also explore the connection and the missing links between NAFLD and extrahepatic cancers. Interestingly, any relationship between NAFLD and extrahepatic malignancies could be attributable to several shared metabolic risk factors. Overall, obesity, insulin resistance, metabolic syndrome, and related disorders may increase the risk of developing cancer. Therefore, early diagnosis of NAFLD is essential for preventing the progression of the disease and avoiding its severe complications. In addition, cancer screening and early detection in these patients may improve survival and reduce any delays in treatment.

## 1. Introduction

Non-alcoholic fatty liver disease (NAFLD) is a term used for a range of liver conditions, including simple steatosis and non-alcoholic steatohepatitis (NASH), which may eventually progress to liver fibrosis, cirrhosis, and cancer [1,2]. Excessive hepatic fat accumulation in patients without significant alcohol consumption represents a major cause of liver dysfunction and chronic liver disease worldwide [3,4,5]. In addition, NAFLD appears to be associated with insulin resistance, metabolic syndrome, diabetes mellitus and obesity [6]. Due to its increasing prevalence, particularly in Western countries, the development of invasive and non-invasive diagnostic tools and the adoption of novel treatment options have been the focus of increased attention in order to improve the prognosis of the disease.

There is a substantially increased risk for overall and liver-related mortality among NAFLD patients [7]. NAFLD is usually a silent liver disease without causing any symptoms. However, it may lead to severe liver-related and extrahepatic complications, such as cardiovascular disease and malignancies [8,9]. To date, accumulated evidence shows a potential association between NAFLD and the incidence of several extrahepatic cancers [10]. As a result, the efforts to better understand the mechanisms linking NAFLD with the risk of developing some malignancies have raised great interest.

Any association between NAFLD and extrahepatic cancers might be attributable to shared metabolic risk factors [11]. The relationship between NAFLD, colorectal cancer (CRC), and adenomatous polyps has been thoroughly investigated during past decades. However, the exact mechanisms correlating NAFLD with CRC, and its precursor lesions, are not entirely understood yet. Furthermore, several researchers have attempted to investigate the association between NAFLD and other extrahepatic neoplasms, including esophageal, gastric, biliary tract, pancreatic and breast cancer [11,12].

A comprehensive literature search in PubMed up to January 2022 was conducted to identify recent studies (mainly during the last 12 years) that analyzed the relationship between NAFLD and different extrahepatic malignancies. We also examined the reference lists of the included articles in order to find additional relevant reports. In fact, the current review focused on all relevant English articles that provided information on the association between patients previously diagnosed with NAFLD and/or extrahepatic cancers.

Our research herein aims to help understand the key issues related to the development and progression of NAFLD. Based on the current literature, CRC is the most well-studied form of cancer observed among NAFLD patients. It also represents one of the most common causes of cancer-related deaths. At first, our objective was to thoroughly examine the relationship between NAFLD, colorectal carcinomas and their precursor lesions. Then, we investigated the association between NAFLD and other extrahepatic malignancies, suggesting the necessity for screening, particularly in cases with high cancer risk. However, further studies are still required in order to shed some light on the role of NAFLD in the above-mentioned forms of cancer. Importantly, NAFLD seems to be an underestimated multisystem disease with far-reaching consequences commonly overlooked by the general population.

## 2. Overview of Non-Alcoholic Fatty Liver Disease (NAFLD)

First described in the 1980s, NAFLD as an umbrella term encompasses a wide range of liver conditions from simple steatosis to NASH, which can progress to liver fibrosis, cirrhosis, and hepatocellular carcinoma [1,2]. Over the past few decades, NAFLD has become an alarming public health concern due to its increasing prevalence, particularly in Western countries, reaching global epidemic proportions in both adults and children [13,14]. Indeed, NAFLD has emerged as a major etiology of chronic liver disease worldwide. It is expected to become the most rapidly growing indication for liver transplantation within the next few years [3].

NAFLD is defined by excessive fat deposition in the liver with the presence of intracellular triglycerides in more than 5% of hepatocytes in patients without significant alcohol consumption (<30 g/day for males, <20 g/day for females) [4,5]. Furthermore, other secondary causes of steatosis, including viral, drug-induced, alcoholic liver disease or acute fatty liver of pregnancy, are excluded [15,16]. As a spectrum of liver disease, NAFLD appears to be closely related to insulin resistance, metabolic syndrome, diabetes mellitus, obesity, and dyslipidemia [6]. Indeed, a growing number of recent studies suggest a significant association between NAFLD, metabolic syndrome and its components [6,17]. However, despite the “chicken-and-egg” conundrum regarding the primacy of either metabolic syndrome over NAFLD or NAFLD over metabolic syndrome, NAFLD is now recognized as the hepatic manifestation of metabolic syndrome [17,18,19].

**Figure 1:**
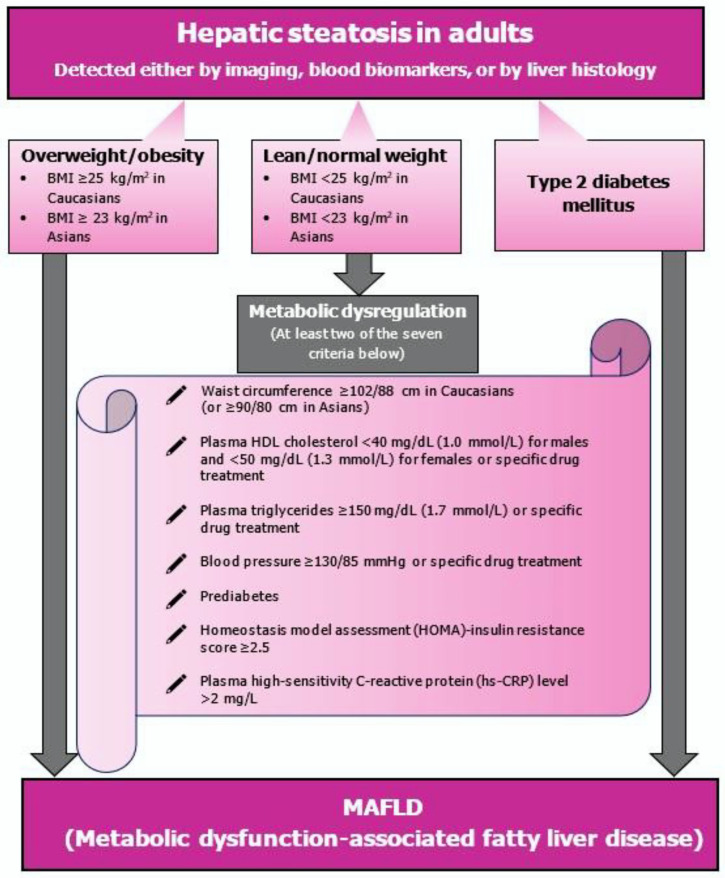
In a bid to raise awareness of the disease, a multidisciplinary group of experts recommended changing the definition and nomenclature of NAFLD to metabolic dysfunction-associated fatty liver disease (MAFLD). MAFLD is diagnosed in patients with steatosis and at least one of the three criteria: obesity/overweight, type 2 diabetes mellitus and any evidence of metabolic dysregulation. NAFLD—non-alcoholic fatty liver disease; MAFLD—metabolic dysfunction-associated fatty liver disease.

At the same time, there have been global efforts to raise awareness of the disease by changing the definition and nomenclature of NAFLD to metabolic dysfunction-associated fatty liver disease (MAFLD). Specifically, an international panel of experts from 22 countries proposed new definition criteria for diagnosing MAFLD, considering the disease heterogeneity and the underlying metabolic factors as the key contributors to the disease progression [20]. MAFLD is diagnosed in patients with evidence of hepatic steatosis (based on imaging, histopathological examination or blood biomarker testing) and the presence of at least one of the following three metabolic criteria: obesity/overweight, established type 2 diabetes mellitus (T2DM) or metabolic dysregulation [20] (Figure 1). The new term reflects a better understanding and knowledge of the underlying pathogenic factors (metabolic dysfunction) that drive MAFLD [20]. NAFLD is, by definition, a diagnosis of exclusion based on negative criteria (i.e., alcohol intake quantification) [20,21]. On the other hand, integrating positive criteria (presence of hepatic steatosis) in the MAFLD definition could reduce confusion on the etiology and stigma of the disease by avoiding any reference to alcohol consumption [20,22]. In fact, adopting the term MAFLD may also lead to abandoning the dichotomous view of NASH and non-NASH [20,22].

### 2.1. Epidemiology and Risk Factors

Overall, the prevalence of NAFLD appears to vary widely depending on the study population (i.e., age, gender, lifestyle, ethnic differences) and the diagnostic methods used [2,23,24,25]. The increasing worldwide prevalence of obesity, T2DM and metabolic syndrome may contribute to the high prevalence of NAFLD in the general population [6,26]. Specifically, the global prevalence of NAFLD is estimated to be around 25%, with the highest rates observed in the Middle East (31.8%) and South America (30.4%), followed by Asia (27.4%), North America (24.1%), and Europe (23.7%). The lowest rate has been registered in Africa (13.5%). Meanwhile, the estimated community prevalence of NASH is approximately 1.5–6.5% [27].

As mentioned previously, there is a close link between NAFLD and obesity. Recent evidence suggests that the distribution of adipose tissue in the body is associated with the metabolic complications of obesity, such as steatosis [6,28,29,30]. NAFLD prevalence may vary between 60% and 95% in the obese population [31,32]. Moreover, among morbidly obese individuals, NAFLD affects 84% to 96% of the patients undergoing bariatric surgery, with severe fibrosis or cirrhosis being present in 2% to 12% of the patients [33,34,35,36]. Obesity is reported in 51% and 81% of patients with NAFLD and NASH, respectively [27]. Furthermore, NAFLD has now become the main driver and leading cause of chronic liver disease among children, mainly due to the rising childhood obesity rates [37,38]. The estimated NAFLD prevalence is 3–10% in the pediatric population, ranging from 50% to 80% among obese and overweight children [39,40].

The increasing rates of T2DM may also lead to increased NAFLD prevalence. Indeed, data extracted from 24 studies involving 35,599 T2DM patients showed a pooled NAFLD prevalence of 59.67% (95% confidence interval [CI], 54.31–64.92), rising to 77.87% (95% CI, 65.51–88.14) in the diabetic patients with obesity [41]. Additionally, in a recent systematic review and meta-analysis of 80 studies involving 49,419 T2DM patients, the estimated global prevalence of NAFLD and NASH among patients with T2DM was 55.5% (95% CI, 47.3–63.7) and 37.3% (95% CI, 24.7–50.0), respectively [42]. Advanced fibrosis was present in 17% (95% CI, 7.2–34.8) of patients with T2DM and NAFLD who underwent liver biopsy [42]. Among NAFLD and NASH patients, the pooled overall prevalence of T2DM is reported to be 22.51% (95% CI, 17.92–27.89) and 43.63% (95% CI, 30.28–57.98), respectively [27].

Furthermore, metabolic syndrome is considered a significant risk factor for developing NAFLD. In a recent cohort study involving 11,674 individuals, the NAFLD prevalence was found to be 43.2% among subjects with metabolic syndrome. Regarding the patients with metabolic syndrome, the prevalence of NAFLD also increased significantly with the number of metabolic syndrome criteria (37%, 49%, and 67% for those patients with three, four and all five criteria, respectively) [43].

Even though NAFLD is closely associated with metabolic syndrome and obesity, it may occur in a proportion of patients who are not obese (known as non-obese or lean NAFLD) [44,45,46,47,48]. Data from the United States suggest that 5% to 10% of NAFLD patients are considered lean (normal body mass index, BMI) [44]. In Europe, approximately 20% of biopsy-proven NAFLD patients are lean [45,46]. Similarly, in Asia, the prevalence rate of lean NAFLD is estimated to be around 19–23% [47,48]. In general, complex interactions between environmental and dietary factors, altered metabolism, and genetic predisposition may eventually lead to the pathogenesis of lean NAFLD [49]; however, further research on this subject is still required.

As noted previously, there are several significant metabolic risk factors for developing NAFLD. Other factors, including age, sex, and ethnicity, also influence the prevalence of NAFLD [50]. In fact, Hispanic ethnicity and advanced age are associated with increased NAFLD prevalence [50]. Higher prevalence rates are also observed in males younger than 50 years and females older than 50 years, probably associated with hormonal changes after menopause [51].

### 2.2. NAFLD Pathogenesis

During the past few decades, significant efforts have been made to clarify the mechanisms underlying NAFLD pathogenesis and progression to NASH. In 1998, Day and James proposed the “two-hit” hypothesis [52]. According to their model of NAFLD pathogenesis, the first hit is represented by intrahepatic triglyceride accumulation triggered mainly by insulin resistance, sedentary lifestyle and central obesity [53,54]. Indeed, insulin resistance is a common cause of increased delivery of free fatty acids and triglycerides to the liver and reduced excretion, eventually leading to intrahepatic fat accumulation [55,56,57,58]. Furthermore, excess carbohydrates represent another significant stimulus for hepatic de novo fatty acid synthesis [55].

The second hit induces hepatocyte inflammation, fibrosis and necrosis [59]. In general, a fatty liver is considered more vulnerable to hepatocellular injury. Increased production of reactive oxygen species, oxidative stress, lipid peroxidation, and mitochondrial dysfunction play a central role in the second hit [55,58,60,61]. Interestingly, insulin resistance and obesity also contribute to the second hit, leading to NASH and fibrosis [58,60,62]. Specifically, adipose tissue may act as a source of inflammatory mediators, releasing adipokines with pro-inflammatory and anti-inflammatory properties, including leptin, interleukin-6 (IL-6), tumour necrosis factor-α (TNF-α), and adiponectin [62,63,64,65,66,67,68]. Several inflammatory pathways are considered to be involved in NAFLD development [69]. In fact, a disturbance in adipokine production may be present in NASH patients (elevated TNF-α levels and lower adiponectin levels) [67,70].

According to the multiple-hit hypothesis, a fatty liver is subject to multiple insults that promote hepatic inflammation and fibrosis [19,71]. These multiple hits include a combination of bad nutritional habits, sedentary lifestyle, insulin resistance, epigenetic modifications, alterations in gut microbiota and adipose tissue dysfunction acting altogether on genetically predisposed individuals [19]. Insulin resistance is one of the critical factors in NAFL/NASH pathogenesis that leads to increased hepatic de novo lipogenesis and adipose tissue dysfunction, producing high levels of circulating free fatty acids [72,73]. At the same time, adipose tissue dysfunction may also result in impaired adipokine and inflammatory cytokine production and secretion [73].

In addition, hepatic fat accumulation could lead to lipotoxicity, a severe condition promoting mitochondrial dysfunction with oxidative stress and further aggravation of endoplasmic reticulum (ER) stress [74]. Oxidative stress is defined by a disrupted balance between reactive oxygen and nitrogen species (ROS and RNS, respectively) and the available antioxidant defense mechanisms [75]. Specifically, oxidative stress plays a critical role by acting as a key modulator in NAFLD pathogenesis [76] and inducing hepatocellular injury, liver inflammation and fibrosis [77]. Recent evidence reveals that mitochondrial dysfunction is a significant contributor to oxidative stress, leading to the overproduction of ROS and the consequent elevation in lipid peroxidation products [78]. It is worth mentioning the fact that mitochondria constitute the primary source of ROS in hepatocytes, as ROS are mostly generated from the endoplasmic reticulum and the mitochondrial electron transport chain [79]. ROS also promote the production of inflammatory cytokines via the activation of nuclear factor-κB (NF-κB) and nucleotide-binding oligomerization domain-like receptor family, pyrin domain-containing protein 3 (NLRP3) pathways [77]. Furthermore, mitochondrial DNA, which is released in the cytoplasm due to mitochondrial oxidative stress, activates Toll-like receptor 9 (TLR9) on Kupffer cells. Activated Kupffer cells promote the activation of hepatic stellate cells, which, if persistent, lead to liver fibrosis [77].

Dietary factors may also play a central role in gut microbiome alterations, increasing intestinal mucosal permeability and bacterial overgrowth, therefore activating inflammatory pathways [80]. The processes mentioned above lead to the development of a chronic inflammatory state accompanied by hepatocellular death, activation of hepatic stellate cells and fibrogenesis [19]. It is currently supported that the timing and the combination of the multiple parallel hits may help determine whether simple steatosis or NASH will be the initial liver lesion [81].

Meanwhile, the I148M point mutation in the patatin-like phospholipase domain-containing protein 3 (PNPLA3) gene represents one of the most significant predisposing factors to NAFLD development and advanced liver damage, indicating that there is also a genomic background in NAFLD pathogenesis [82,83,84,85]. In fact, it has been suggested that the accumulation of PNPLA3-148M on the surfaces of lipid droplets (LDs) inhibits triacylglycerol hydrolysis and leads to the impaired mobilization of triglycerides from LDs [86,87,88].

### 2.3. Histological Features

**Figure 2:**
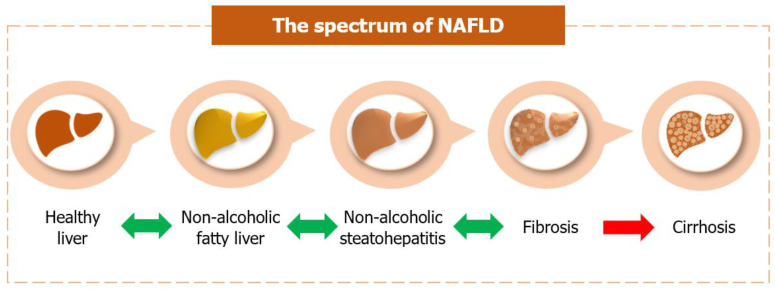
Non-alcoholic fatty liver: There is fat accumulation within hepatocytes at this stage, a process known as hepatic steatosis. Non-alcoholic steatohepatitis: The accumulation of fat in the hepatocytes is accompanied by liver inflammation and hepatocellular ballooning. Fibrosis: Fibrotic scar tissue starts to form in an inflamed liver. According to the NASH Clinical Research Network (CRN) scoring system, fibrosis staging includes stage 0 (no fibrosis), stage 1A (mild perisinusoidal fibrosis), stage 1B (moderate perisinusoidal fibrosis), stage 1C (portal/periportal fibrosis), stage 2 (perisinusoidal and portal/periportal fibrosis), stage 3 (bridging fibrosis), and stage 4 (cirrhosis). Cirrhosis: End-stage liver disease, in which the formation of fibrotic septa bridges together adjacent portal tracts and central veins. There is an increased risk of hepatocellular carcinoma development. NASH—non-alcoholic steatohepatitis; CRN—Clinical Research Network.

Hepatocellular steatosis (usually macrovesicular) is usually observed in patients with non-alcoholic fatty liver [89]. Other histopathological abnormalities required for the NASH diagnosis include steatosis (macro > micro), lobular inflammation and hepatocellular ballooning (typically seen in the zone 3 steatotic liver cells of the adolescent and adult patients) with or without perisinusoidal fibrosis [89]. Younger children may appear with an alternate pattern characterized by a prominent distribution of steatosis and inflammation in zone 1 [89]. Additionally, Mallory-Denk bodies (eosinophilic cytoplasmic protein aggregates), megamitochondria, hepatocellular glycogenated nuclei, acidophil bodies (apoptotic hepatocytes), and iron deposition represent common histopathological findings in NASH [90]. In most cases, simple steatosis is generally considered to follow a relatively benign clinical course [91]. However, NASH is associated with a more progressive disease course, leading to advanced fibrosis or cirrhosis with all its consequences [92] (Figure 2). In fact, severe NASH may eventually progress to “burnt-out” cirrhosis, for which no characteristic histopathological features remain [15].

### 2.4. Clinical Features and Diagnostic Workup

Regarding the symptoms, NAFLD is often clinically silent. However, if present, most symptoms are usually non-specific, such as right upper quadrant discomfort or pain (sharp/dull quality), fatigue, abdominal bloating, and sleep disturbances [93]. Mild or moderate hepatomegaly may be present in some NAFLD cases on physical examination.

Most subjects with NASH-related cirrhosis and end-stage liver disease could present with nausea, jaundice, pruritis, ascites, memory impairment and anorexia. Furthermore, end-stage liver disease clinical signs include jaundice, palmar erythema, spider angiomas, caput medusae, ascites, Dupuytren contracture, and petechiae [94].

According to recent research, several studies support that patients with NAFLD are more prone to develop liver injury and experience more severe symptoms when infected with coronavirus disease-2019 (COVID-19) caused by severe acute respiratory syndrome coronavirus 2 (SARS-CoV-2) [95,96]. However, other studies report no significant difference in the clinical course between COVID-19 patients with and without fatty liver [96,97]. Indeed, further studies are required to gain a better knowledge of the interactions between COVID-19 and other diseases, such as NAFLD.

Liver biopsy remains the gold standard for diagnosing NAFL/NASH [98]. Indeed, this technique represents an essential tool for histologic evaluation, as it provides information regarding hepatic steatosis, hepatocellular injury, inflammation and fibrosis [98]. Another sensitive tool used for assessing the changes in NAFLD patients during therapeutic trials is the NAFLD activity score (NAS) [99,100]. The steatosis, activity, and fibrosis (SAF) scoring system is a sum of scores used to evaluate the histological severity of NAFLD [98,101,102]. However, none of these histological grading and staging systems for the disease were formed to replace the diagnostic determination of NAFLD [99]. In general, a few significant pathological classifications have been suggested for NAFLD, including NAS, Matteoni’s classification, and Brunt’s classification [100,103,104]. Liver biopsy is an invasive technique with several drawbacks, such as postprocedural complications, sampling error and high costs [105,106,107]. The experience of the pathologists, inter-observer, and intra-observer variability also represent critical success factors [108,109,110,111]. Therefore, this method should be limited to subjects with diagnostic uncertainty or indeterminate non-invasive staging [112].

Such limitations have raised great interest in developing non-invasive approaches for NAFL/NASH diagnosis. These diagnostic imaging techniques besides ultrasonography (US) include magnetic resonance imaging (MRI) and computed tomography (CT) [113,114]. Additionally, vibration-controlled transient elastography (VCTE) is an essential non-invasive approach for evaluating fibrosis and steatosis in NAFLD patients by measuring liver stiffness [115]. MRI with elastography (MRE) also constitutes a novel technique with high diagnostic accuracy in staging liver fibrosis among NAFLD patients [116,117,118]. Other methods include controlled attenuation parameter (CAP) and acoustic radiation force impulse (ARFI), which are used to assess hepatic steatosis and fibrosis, respectively [112,119].

Interestingly, laboratory testing may be normal in NAFLD. However, in some cases, alanine aminotransferase (ALT), aspartate aminotransferase (AST), alkaline phosphatase (ALP), gamma-glutamyl-transpeptidase (GGT), serum ferritin or triglyceride levels may be increased [120]. Meanwhile, several noninvasive scoring systems are used to assess liver fibrosis, such as the AST to platelet ratio index (APRI), the AST/ALT ratio, the BARD score (BMI ≥ 28, AST/ALT ratio ≥ 0.8 and diabetes mellitus), the fibrosis-4 (FIB-4) score (age, AST, platelet count, ALT), the NIKEI (age, AST, total bilirubin and the AST/ALT ratio), the Enhanced Liver Fibrosis score (tissue inhibitor of metalloproteinase-1, hyaluronic acid and type III procollagen peptide), the FibroMeter NAFLD score (age, body weight, platelet count, AST, ALT, ferritin and glucose), and the NAFLD fibrosis score (age, BMI, platelet count, impaired fasting glucose/diabetes, serum albumin and the AST/ALT ratio) [121]. In addition, FibroTest includes several parameters, such as GGT, total bilirubin, alpha-2-macroglobulin, apolipoprotein A1, and haptoglobin, adjusted for the patient’s age and gender, while ActiTest includes the same biomarkers plus ALT [122]. Furthermore, practical algorithms for predicting hepatic steatosis are the following:

- Fatty liver index (waist circumference, BMI, triglycerides and GGT) [123,124],
- Hepatic steatosis index (BMI, gender, diabetes and the AST/ALT ratio) [125],
- Lipid accumulation product (waist circumference and triglycerides) [126],
- Triglyceride-glucose (TyG) index (fasting glucose and triglyceride levels) [127],
- Visceral adiposity index (waist circumference, BMI, triglycerides and high-density lipoprotein cholesterol levels) [128].

Promising biomarkers, which are related to NASH and may help differentiate steatosis from steatohepatitis, are cytokeratin-18 (CK-18) [129], the terminal peptide of procollagen III (PIIINP) [130], IL-6 [131], TNF-α [132], the chemokines MCP-1 and RANTES [133], and fibroblast growth factor 21 (FGF21) [134]. In addition, the single nucleotide polymorphisms (SNPs) rs738409 and rs58542926 could also be promising genetic markers for NAFLD progression and assessment of hepatic steatosis, respectively [135,136,137,138]. However, further validation of the biomarkers mentioned above is still required. Currently, several studies suggest the use of asymmetric dimethylarginine (ADMA) and microparticles released by damaged hepatocytes as potential biomarkers for NAFLD and NASH diagnosis, respectively [139,140,141]. Based on liquid chromatography and mass spectrometry, Verdam et al. [142] suggested that NASH diagnosis could also be achieved by analyzing volatile organic compounds in the exhaled breath.

### 2.5. Treatment

Therapeutic efforts should focus not only on the liver disease itself, but also on its related metabolic disorders. At present, lifestyle modifications, including a healthy diet, weight loss, and increased physical activity, may help improve NAFLD and control blood glucose, pressure, triglycerides, and high-density lipoprotein (HDL) cholesterol levels [143,144]. Indeed, recent studies showed that a healthy diet and exercise could improve liver fat as assessed by US and MRI among NAFLD patients [145,146]. Another important meta-analysis found that patients with a ≥5% weight reduction had significant improvements in hepatic steatosis [147]. In addition, a weight loss of ≥7% was associated with improved histological disease activity (NAS) [147]. Several studies have also shown the positive effects of the combination of low-carbohydrate and Mediterranean diets on body weight and hepatic fat content [148,149]. Meanwhile, bariatric surgery represents another approach for weight reduction and should be considered in selected cases (BMI ≥40 kg/m^2^ or a BMI of 35–39.9 kg/m^2^ with at least one comorbidity) [150,151]. Bariatric procedures lead to weight loss, reduced chronic inflammation related to obesity, and significant improvements in lipid metabolism and insulin tolerance [152,153].

At first, vitamin E was found to improve liver function and histological findings in NASH patients [154,155]. As insulin resistance plays a vital role in the pathogenesis of NAFLD, several anti-diabetic agents have been also evaluated for their efficacy in treating NAFL/NASH. Pioglitazone administration is suggested for NASH patients with insulin resistance [154,155,156]. Sodium-glucose co-transporter 2 (SGLT2) inhibitors improve liver enzymes, glucose metabolism and histological findings in NAFL/NASH patients with T2DM, and thus its administration is recommended [15,157,158,159]. In addition, incretin-related drugs, such as glucagon-like peptide-1 (GLP-1) receptor analogue and dipeptidyl peptidase-4 (DPP-4) inhibitor, improve liver function, glucose metabolism and histological findings in NAFLD patients with T2DM [15,160,161,162,163,164,165].

Furthermore, 3-hydroxy-3-methylglutaryl-coenzyme A (HMG-CoA) reductase inhibitors are suggested for patients with NAFL/NASH and hypercholesterolemia [15,166,167,168,169,170]. It is also worth mentioning that the effects of DPP-4 and ezetimibe are not constant [15]. Overall, angiotensin II receptor blockers (ARBs) and angiotensin-converting enzyme (ACE) inhibitors are the recommended treatments for NASH patients with hypertension [15]. At the same time, there is no evidence indicating any improvement in liver histology through the administration of ursodeoxycholic acid and biguanides among NAFL/NASH patients [171,172,173]. Lastly, multiple ongoing trials are targeting different pathways in the NAFL/NASH pathogenesis, such as trials of elafibranor, obeticholic acid, selonsertib, cenicriviroc, emricasan, pemafibrate, apoptosis signal-regulating kinase 1, aramchol, Toll-like receptor 4 inhibitor, fibroblast growth factor 21, acetyl-CoA carboxylase inhibitor, heat shock protein 47 and galectin-3 inhibitor [15].

## 3. Association between NAFLD and Extrahepatic Cancers

**Figure 3:**
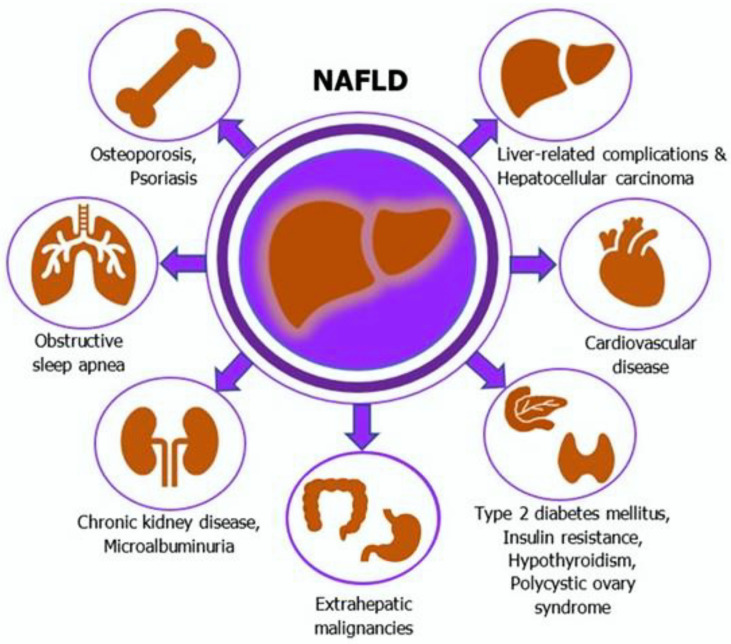
NAFLD is a multisystem disease leading to severe liver-related and extrahepatic complications. NAFLD—non-alcoholic fatty liver disease.

Over the last decade, it has been shown that there is a substantially increased risk for overall and liver-related mortality in patients with NAFLD [7]. In fact, convincing evidence suggests that NAFLD is a multisystem disease and potentially leads to a significant burden of severe liver-related and extrahepatic complications, including cardiovascular disease, T2DM, and malignancies [8,9] (Figure 3). Current findings reveal increased incidence rates of several extrahepatic malignancies among NAFLD patients [10]. However, the exact mechanisms of how NAFLD could be associated with an increased risk of developing some cancers are not completely understood. A summary of recent studies investigating the association between NAFLD and colorectal neoplasms is presented in Table 1 [174,175,176,177,178,179,180,181,182,183,184,185,186,187,188,189,190,191,192,193,194,195,196,197,198,199,200,201,202,203,204,205,206].

### 3.1. NAFLD and Colorectal Adenomas

Several studies have attempted to investigate the association between NAFLD and colorectal adenomatous polyps, but the reported results remain quite controversial. Nevertheless, most authors demonstrated that NAFLD is significantly associated with an increased risk of developing colorectal adenomas [174,176,177,178,180,184,185,186,187,188,189,191,193,195,196,199,200,203,205]. In 2010, a cross-sectional study by Hwang et al. [174] analyzed 2917 participants who underwent a routine colonoscopy based on the evaluation of abdominal ultrasonography, different anthropometric measurements, and other laboratory panels such as liver function tests. The estimated prevalence of NAFLD was 41.5% and 30.2% in the polyp and control groups, respectively. In addition, NAFLD was correlated with a high risk of developing colorectal adenomatous lesions (odds ratio (OR), 1.28; 95% CI, 1.03–1.60; *p* = 0.029). The authors also showed that the increased risk for NAFLD was primarily observed in patients with multiple adenomas in the polyp group.

Touzin et al. [175] retrospectively analyzed 233 patients who underwent screening colonoscopy, and liver biopsy or abdominal ultrasound. They found no significant difference between the NAFLD and the control groups in the prevalence of colonic adenomas (*p* = 1.00). However, a low polyp burden was noted in patients with negative ultrasounds for NAFLD. In a cross-sectional study, Wong et al. [176] analyzed 380 community and consecutive patients who underwent a screening colonoscopy. The diagnosis of NAFLD was achieved through proton-magnetic resonance spectroscopy or liver biopsy. The prevalence of colorectal adenomas was higher in NAFLD patients compared with the controls (34.7% vs. 21.5%; *p* = 0.043). Among the biopsy-proven NAFLD patients, the prevalence of colorectal adenomatous polyps was also higher in the NASH group compared with the NAFL group (51% vs. 25.6%; *p* = 0.005). At the same time, NASH was found to be significantly associated with the presence of colorectal adenomas (adjusted OR, 4.89; 95% CI, 2.04–11.70; *p* < 0.001).

Huang et al. [180] conducted a study on 1522 participants who underwent two consecutive colonoscopies. The NAFLD prevalence was higher in the adenoma group, but not in the non-adenoma group subjects (55.6% vs. 38.8%; *p* < 0.05). The authors revealed that NAFLD was found to be an independent risk factor for developing colorectal adenomas following an initial negative baseline colonoscopy (OR, 1.45; 95% CI, 1.07–1.98; *p* = 0.016). The risk of developing colorectal adenomatous polyps was increased in NAFLD patients, particularly when other comorbidities were present. In 2015, Bhatt et al. [184] retrospectively studied 591 patients who completed the liver transplant evaluation process. The prevalence of colorectal polyps and adenomas was higher in the NAFLD group compared with the non-NAFLD group (59% vs. 40%; *p* = 0.003, and 32% vs. 21%; *p* = 0.04, respectively). The presence of NAFLD appeared to be a significant predictor of finding a colorectal polyp and adenoma (adjusted OR, 2.42; 95% CI, 1.42–4.11; *p* = 0.001, and adjusted OR, 1.95; 95% CI, 1.09–3.48; *p* = 0.02, respectively).

In addition, Chen and colleagues [188] analyzed 3686 individuals who underwent abdominal ultrasound and colonoscopy as part of a health check-up program. They reported that NAFLD was independently associated with an increased risk of developing colorectal polyps and adenomas (adjusted OR, 1.26; 95% CI, 1.05–1.51; *p* < 0.05, and adjusted OR, 1.28; 95% CI, 1.01–1.64; *p* < 0.05, respectively). Furthermore, after sex analysis, the researchers observed a significant association between NAFLD and adenomas in men (adjusted OR, 1.53; 95% CI, 1.18–2.00; *p* < 0.05), but not in women. NAFLD was also correlated with the presence of multiple colorectal adenomas (OR, 1.82; 95% CI, 1.29–2.55; *p* = 0.001), distal adenomas (OR, 1.63; 95% CI, 1.11–2.39; *p* = 0.013), and bilateral adenomas (OR, 1.89; 95% CI, 1.23–2.91; *p* = 0.004). In a study by Ze et al. [191], the authors noted that fatty liver index (FLI) ≥ 30 was associated with a high risk of developing colorectal adenomas (OR, 1.26; 95% CI, 1.06–1.49; *p* = 0.008). In fact, patients with FLI ≥ 30 presented with a greater frequency of multiple or advanced adenomas than those with FLI < 30.

In another study, Kim et al. [193] analyzed 6332 subjects who underwent abdominal ultrasound and 1st-time colonoscopy. According to their results, NAFLD was found to be an independent risk factor for colorectal adenomas (adjusted OR, 1.15; 95% CI, 1.02–1.30; *p* = 0.027), advanced (adjusted OR, 1.50; 95% CI, 1.12–2.01; *p* = 0.006) and multiple (adjusted OR, 1.32; 95% CI, 1.01–1.73; *p* = 0.006) adenomas. Recently, Cho et al. [196] found that NAFL and NASH were separately associated with an increased risk of developing polyps (adjusted OR, 2.76; 95% CI, 1.51–5.06; *p* = 0.001, and adjusted OR, 2.08; 95% CI, 1.12–3.86; *p* = 0.02, respectively).

In another study by Blackett et al. [199], the researchers also observed that the prevalence of colorectal adenomas was significantly increased in the NAFLD group compared with the control group (40.7% vs. 28.1%; *p* = 0.01). However, the risk of developing adenomas was not correlated with the severity of NAFLD, particularly with the presence or not of steatohepatitis (adjusted OR, 2.47; 95% CI, 0.67–9.1; *p* = 0.17). Meanwhile, Yu et al. [201] conducted a cross-sectional study by analyzing 1538 patients with colorectal polyps who underwent abdominal ultrasound and colonoscopy. There was no significant difference in the location and morphology of the polyps between the NAFLD and the control groups (*p* > 0.05). In fact, NAFLD was correlated with the detection of colorectal polyps, especially among patients with multiple polyps, those with a large size and villous features (*p* < 0.05).

At present, Fukunaga et al. [203] studied 124 consecutive health check-up examinees who underwent a colonoscopy. They found a significant association between colorectal adenomas and MAFLD, mainly non-obese MAFLD. Furthermore, in a retrospective cohort study, Seo et al. [205] supported that NAFLD and MAFLD were significantly associated with an increased risk of developing adenomas in women (adjusted OR, 1.43; 95% CI, 1.01–2.03; *p* = 0.046, and adjusted OR, 1.55; 95% CI, 1.09–2.20; *p* = 0.015, respectively).

As mentioned in the above studies, NAFLD patients exhibit an increased risk of developing colorectal adenomas, particularly multiple polyps, most commonly localized in the right and transverse segments of the colon [11,184]. Interestingly, the relationship between NAFLD and colorectal adenomatous polyps emphasizes the necessity of closer surveillance for the early detection of colorectal cancer. However, further evidence is still required to find the ideal target group for the CRC screening of NAFLD patients. It is crucial to evaluate and determine the appropriate age range for CRC screening among NAFLD patients based on results from larger population studies.

### 3.2. NAFLD and Colorectal Cancer

The relationship between NAFLD, CRC and its precursor lesions has been extensively investigated during the past few decades. In general, most guidelines recommend that regular screening should start at age 45 [207]. Obesity, cigarette smoking, and increased alcohol consumption may also be considered significant risk factors for CRC development [208]. In fact, current findings suggest that metabolic syndrome and eventually NAFLD, which is the liver manifestation of metabolic syndrome, may also increase the risk of developing colorectal carcinomas [208]. These associations may guide us to perform screening colonoscopy earlier or more frequently in patients with metabolic syndrome or NAFLD [177,209].

In 2011, a cross-sectional study by Wong et al. [176] analyzed 380 community and consecutive patients undergoing screening colonoscopy. The prevalence of advanced colorectal neoplasms was found to be 18.6% in the NAFLD group and 5.5% in the control group (*p* = 0.002). Regarding the biopsy-proven NAFLD patients, the prevalence of advanced colorectal neoplasms was 34.7% in the NASH group and 14% in the NAFL group (*p* = 0.011). In fact, NASH was significantly associated with the development of advanced colorectal neoplasms (OR, 5.34; 95% CI, 1.92–14.84; *p* = 0.001). Furthermore, Stadlmayr et al. [177] conducted a study on 1211 patients undergoing screening colonoscopy and observed a higher risk of developing CRC in NAFLD patients. The CRC prevalence in men was significantly increased in the NAFLD group than in the control group (1.6% vs. 0.4%; *p* < 0.001).

In another research, Min et al. [179] retrospectively analyzed 227 patients diagnosed with CRC. They found no significant difference between CRC patients with and without NAFLD regarding the location and differentiation of tumors, carcinoembryonic antigen (CEA), and the total number of synchronous or advanced adenomas. Moreover, the presence of NAFLD did not influence CRC prognosis. Nevertheless, CRC patients with NAFLD were diagnosed earlier compared with CRC patients without NAFLD (*p* = 0.004). Lin et al. [181] conducted a retrospective and consecutive cohort study on 2315 community participants undergoing a routine colonoscopy. NAFLD appeared to be an independent risk factor for developing colorectal malignant neoplasms (OR, 1.868; 95% CI, 1.360–2.567; *p* = 0.001).

Another study by You et al. [182] retrospectively analyzed 1314 CRC patients who underwent surgical resection of the tumor. They noted no significant difference in disease-free survival (DFS) rates between CRC patients with NAFLD and those without NAFLD (*p* = 0.267). In fact, after the adjustment for different clinicopathologic covariates, NAFLD was revealed to be an independent negative risk factor for overall survival (OS) (hazard ratio, 0.593; 95% CI, 0.442–0.921; *p* = 0.02), but not for DFS (*p* = 0.270). In addition, Basyigit et al. [183] studied 127 consecutive patients who underwent colonoscopy and found that CRC prevalence was significantly higher in patients with insulin resistance (*p* = 0.005). However, the risk of developing CRC was increased in patients with insulin resistance, but without NAFLD (OR, 5.218; 95% CI, 1.538–7.448; *p* = 0.017).

In a cross-sectional study, Lee et al. [185] analyzed 44,220 individuals participating in a health check-up program and found that the risk of developing colorectal neoplasms increased with worsening fatty liver severity. Currently, Pan et al. [186] also observed a significant association between the presence of CRC and NAFLD (adjusted OR, 2.164; 95% CI, 1.289–3.217; *p* = 0.005).

Ahn and colleagues [187] found a significant correlation between NAFLD and colorectal neoplasia (adjusted OR, 1.21; 95% CI, 0.99–1.47; *p* = 0.053). The researchers supported that the risk of developing advanced neoplasms appeared significantly higher for patients with severe liver diseases. Furthermore, Yang et al. [189] studied 1023 patients who had previously undergone surveillance colonoscopy following an index colonoscopy. In fact, at 3 and 5 years after the patients’ index colonoscopy, the overall colorectal neoplasm occurrence was 9.1% vs. 5% (NAFLD group vs. non-NAFLD group), and 35.2% vs. 25.3% (NAFLD group vs. non-NAFLD group), respectively (*p* = 0.01). Even though NAFLD was independently correlated with an increased risk of developing colorectal neoplasms, it was not associated with the presence of advanced colorectal neoplasms (adjusted hazard ratio, 1.07; 95% CI, 0.51–2.26; *p* = 0.85).

In another study, Kim et al. [190] analyzed 25,947 individuals who underwent colonoscopy as part of a screening program. NAFLD was significantly associated with colorectal carcinomas in men (adjusted hazard ratio, 2.01; 95% CI, 1.10–3.68; *p* = 0.02), but not in women. Additionally, the severity of NAFLD was not correlated with CRC development. Recently, Chen et al. [192] conducted a cross-sectional study and observed that significant NAFLD was an independent risk factor for CRC-specific mortality in women.

Hamaguchi et al. [194] analyzed 15,926 individuals participating in a health check-up program and found that NAFLD with obesity was independently associated with an increased risk of developing CRC (adjusted hazard ratio, 2.96; 95% CI, 1.44–6.09; *p* = 0.003). Although Cho et al. [196] noted an association between NAFLD and colorectal adenomas, no significant association was observed between NAFLD and advanced colorectal neoplasms. Nevertheless, NASH was independently associated with an increased risk of developing advanced colorectal neoplasms (adjusted OR, 2.81; 95% CI, 1.01–7.87; *p* = 0.049).

In a retrospective cohort study, Lee et al. [198] studied 8,120,674 subjects who received healthcare check-ups. NAFLD (FLI ≥ 60) was correlated with an increased risk of developing colon cancer (hazard ratio, 1.23; 95% CI, 1.19–1.26) and all-cause mortality in CRC patients (hazard ratio, 1.16; 95% CI, 1.10–1.22). To date, Blackett et al. [199] found no significant association between NAFLD and the risk of developing advanced neoplastic lesions (adjusted OR, 2.2; 95% CI, 0.93–5.18; *p* = 0.07).

Kim et al. [204] mentioned a significant association between NAFLD and an increased risk of developing metachronous overall colorectal neoplasia in males (adjusted hazard ratio, 1.17; 95% CI, 1.06–1.29) and females (adjusted hazard ratio, 1.63; 95% CI, 1.27–2.07). Furthermore, an association was also observed between NAFLD and metachronous advanced colorectal neoplasia in women (adjusted hazard ratio, 2.61; 95% CI, 1.27–5.37). Finally, Lee et al. [206] supported that fatty liver disease was correlated with a high risk of developing CRC. The CRC risk was significantly increased in cases with MAFLD, particularly when accompanied by liver fibrosis.

As stated in previous studies, NAFLD patients undergoing a screening colonoscopy were diagnosed with CRC earlier than individuals without NAFLD [176,210]. Although a causal connection between NAFLD and CRC cannot be confirmed, the results of these studies suggest a moderately increased prevalence of CRC among NAFLD patients [210]. Still, further research is required to evaluate the benefits of earlier screening colonoscopy and the role of NAFLD as a predictor for the development of CRC.

### 3.3. Pathophysiological Links between NAFLD, Colorectal Adenomas and Cancer

The association between NAFLD and colorectal neoplasms is the most extensively analyzed in current literature. Nevertheless, the exact pathological mechanisms underlying the link between NAFLD, CRC and colorectal adenomas are not fully understood yet. Considering the bidirectional relationship and strong association between NAFLD and metabolic syndrome [11], several researchers proposed insulin resistance as a significant factor in promoting colorectal neoplasms’ development [177]. Indeed, low-grade chronic inflammation [10,174,211,212] in combination with insulin resistance could create a specific microenvironment that would play a key role in cancer initiation and growth via the stimulation of the insulin growth factor-1 (IGF-1) axis by hyperinsulinemia [11]. This pathway may promote tumorigenesis through its anti-apoptotic and proliferative effects [11]. Such effects are also observed through the up-regulation of leptin/AMP-activated protein kinase and resistin/nuclear factor kappa-light-chain-enhancer of activated β cells [NF-κB], the downregulation of adiponectin/caspase or the activation of TNF-α [11]. It should also be kept in mind that adipose tissue dysfunction may represent another possible mechanism leading to cancer development. NAFLD patients appear to have low adiponectin levels and high leptin levels. Overall, adiponectin is considered to have anticarcinogenic effects. It inhibits colorectal cancer cell proliferation via the cyclic AMP-activated protein kinase and induces the caspase-dependent pathway endothelial cell apoptosis [11]. Additionally, adiponectin inhibits TNF-α, which is implicated in the processes of tumor cell proliferation and angiogenesis [11].

On the other hand, leptin has been shown to increase cancer cell invasiveness by activating the mitogen-activated protein kinase (MAPK) pathway in human colon cancer cells [11,213,214]. The state of chronic low-grade inflammation related to insulin resistance contributes to the build-up of a microenvironment favorable to the development of neoplasms. High levels of proinflammatory cytokines increase cellular proliferation and trigger the inhibition of apoptosis and angiogenesis [10,174]. Although animal studies have supported a causal relationship between alterations in gut microbiota and NAFLD, few human studies have started to describe the presence of such alterations among NAFLD patients [215]. Recently, it has been suggested that microRNAs (miR) (particularly miR-21 and miR-451 acted as an oncogene and a tumor suppressor gene, respectively) may play an essential role during NAFLD and CRC development [216,217]. However, further studies are required to establish a causal relationship between microRNAs and the development of NAFLD and CRC.

### 3.4. NAFLD and Other Extrahepatic Cancers

**Figure 4:**
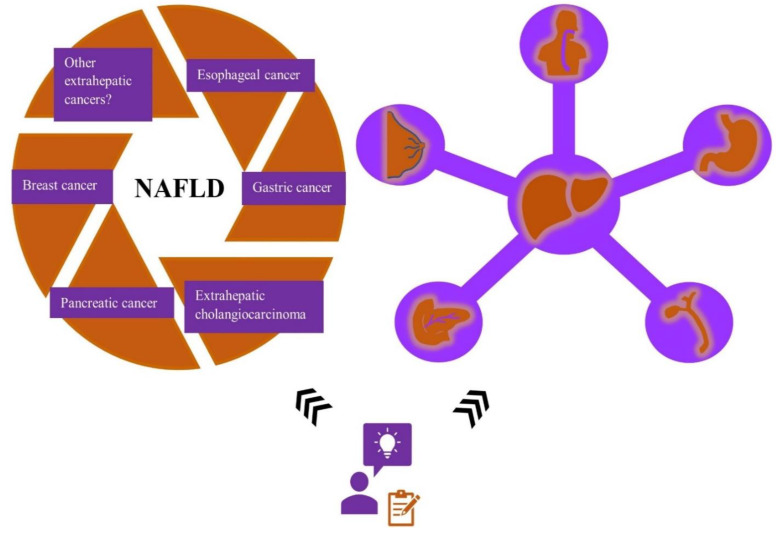
The association between NAFLD and the risk of developing other extrahepatic malignancies besides colorectal cancer remains a subject of ongoing research. Recent studies suggest that NAFLD and metabolic syndrome might be closely related to an increased cancer risk. NAFLD—non-alcoholic fatty liver disease.

During the past decades, several researchers examined the influence of obesity, particularly abdominal fatness, on the risk of developing various extrahepatic cancers. Overall, abdominal obesity is strongly associated with metabolic syndrome. Consequently, the hepatic manifestation of metabolic syndrome, NAFLD, and its correlation with cancer risk is now considered a topic of great interest and ongoing research. Whether NAFLD could lead to an increased risk of developing cancer is still a subject of heated debate. However, more recent studies focused on exploring the role of NAFLD in extrahepatic malignancies to identify if NAFLD could act as a driving force in cancer development (Figure 4).

Esophageal cancer, the 8th most common malignancy globally, is considered to be strongly associated with obesity [11]. In fact, obesity represents a significant risk factor for developing esophageal cancer by increasing the risk up to approximately four-fold compared with lean individuals [11,218]. Several studies support that central adiposity, independent of BMI, is associated with the development of Barrett’s esophagus (BE) and esophageal adenocarcinoma (EAC), eventually contributing to the progression from inflammation to metaplasia (BE) and neoplasia (EAC) [219]. According to other researchers, their results showed a significant dose-dependent correlation between BMI and the risk of developing EAC [218]. Subjects with higher waist circumferences, independent of BMI, were found to be at a 1.5–2.8-fold increased risk of developing BE among both males and females [220]. Similarly, other studies also suggest that visceral abdominal fat could represent a significant risk factor for the development of BE [221].

Despite the potential association between obesity and esophageal cancer, visceral obesity is also closely related to metabolic dysregulation and NAFLD [222,223]. Considering these findings, more researchers focused on investigating the relationship between NAFLD and esophageal cancer. In 2017, Kim et al. [190] observed that a high NAFLD fibrosis score and FIB-4 score were strongly associated with the development of several cancers, including cancer of the esophagus. However, there was no significant difference in the incidence of esophageal cancer between patients with and without NAFLD. Contrary to their expectations, Allen et al. [197] also observed that obese or NAFLD patients did not exhibit an increased risk of developing esophageal cancer. Recently, Lee et al. [198] noted that NAFLD (FLI score ≥ 60) was significantly correlated with a high risk of developing esophageal cancer (hazard ratio, 2.10; 95% CI, 1.88–2.35) and all-cause mortality in esophageal cancer patients (hazard ratio, 1.46; 95% CI, 1.28–1.67).

In a current study, Hamaguchi et al. [194] found that NAFLD with obesity was a significant risk factor for developing gastric cancer (adjusted hazard ratio, 3.58; 95% CI, 1.73–7.38; *p* = 0.001). Furthermore, Allen et al. [197] revealed that the highest risk of malignancy among NAFLD patients was observed in liver cancer, followed by uterine and gastric cancer (incidence rate ratio, 2.3; 95% CI, 1.3–4.1). In agreement with these results, Lee et al. [198] showed that NAFLD was associated with an increased risk of developing gastric cancer (hazard ratio, 1.18; 95% CI, 1.14–1.22) and all-cause mortality in gastric cancer patients (hazard ratio, 1.26; 95% CI, 1.18–1.34). Hence, another critical issue that must be addressed is whether NAFLD patients should be encouraged to undergo screening for gastric cancer.

In a recent meta-analysis involving six studies and after trial sequential analyses, Corrao et al. [224] concluded that NAFLD was significantly associated with intrahepatic cholangiocarcinoma, but not with extrahepatic cholangiocarcinoma. Petrick et al. [225] found that NAFLD was correlated with nearly three-times the risk of developing intrahepatic cholangiocarcinoma (OR, 3.52; 95% CI, 2.87–4.32; *p* < 0.0001) and extrahepatic cholangiocarcinoma (OR, 2.93; 95% CI, 2.42–3.55; *p* < 0.0001). In another study, the researchers observed that NASH was a risk factor for intrahepatic cholangiocarcinoma, eventually affecting its prognosis [226]. Finally, conducting a cohort study, Park et al. [227] showed a significant association between NAFLD and the risk of developing biliary tract cancer (adjusted hazard ratio, 1.28; 95% CI, 1.20–1.37), including cholangiocarcinoma (adjusted hazard ratio, 1.33; 95% CI, 1.23–1.43) and gallbladder cancer (adjusted hazard ratio, 1.14; 95% CI, 1.003–1.29). The adjusted hazard ratios for biliary tract cancer risk tended to increase progressively with the increasing FLI (*p* for trend < 0.001).

Some researchers investigated the relationship between BMI, abdominal fatness, and pancreatic cancer risk [228]. Indeed, there was a significant association between BMI and waist circumference with the risk of developing pancreatic cancer [228]. In addition, when the analyses were restricted to nonsmokers, there was an increased risk of pancreatic cancer development even among individuals within the normal BMI range [228]. Another study revealed a significant correlation between metabolic syndrome and pancreatic cancer (relative risk, 1.58; *p* < 0.0001). This association was stronger in females than in males (*p* = 0.01) [229].

In contrast to Kim et al. [190], who did not observe any difference in the incidence of pancreatic cancer between subjects with and without NAFLD, Chang et al. [230] revealed a positive correlation between NAFLD and pancreatic cancer risk. In fact, pancreatic cancer patients with NAFLD had poorer overall survival than patients without NAFLD, suggesting that NAFLD could be used as a prognostic factor for pancreatic cancer. Allen et al. [197] also observed an increased risk of developing pancreatic cancer among NAFLD patients (incidence rate ratio, 2.0; 95% CI, 1.2–3.3), particularly at a younger age (incidence rate ratio, 0.85; 95% CI, 0.74–0.98).

In 2017, Lee et al. [231] supported that the NAFLD prevalence among breast cancer patients did not differ from that of the general population. Meanwhile, contrary to Lee et al., current studies have shown a correlation between NAFLD and breast cancer. At first, Nseir et al. [232] found that the NAFLD prevalence was higher in females with breast cancer compared with the control group (45.2% vs. 16.4%, *p* = 0.002). Multivariate analysis revealed a significant association between NAFLD and breast cancer (OR, 2.82; 95% CI, 1.2–5.5; *p* = 0.016). Then, Kim et al. [190] also noted a strong association between NAFLD and breast cancer in females (hazard ratio, 1.92; 95% CI, 1.15–3.20; *p* = 0.01). In another research, Kwak et al. [233] observed the correlation between NAFLD and breast cancer in the nonobese subjects (OR, 3.04; 95% CI, 1.37–4.32; *p* = 0.002), but not in the obese subjects (*p* = 0.163).

In addition, breast cancer patients with NAFLD showed a poorer prognosis for tumor recurrence than patients without NAFLD [234]. Allen et al. [197] did not find any correlation between breast cancer risk and NAFLD. However, Park et al. [235] revealed a significant association between the FLI scores (of 30–60 and ≥60) and breast cancer in postmenopausal women (hazard ratio, 1.07; 95% CI, 1.04–1.11, and hazard ratio, 1.11; 95% CI, 1.05–1.17, respectively), but not in premenopausal ones. Currently, Huber et al. [236] supported that NAFLD was a significant risk factor for developing breast cancer in females (hazard ratio, 1.2; 95% CI, 1.01–1.43; *p* = 0.036).

The association between NAFLD and other extrahepatic malignancies is less frequently reported and proven. A meta-analysis conducted by Maclnnis et al. [237] revealed a weak association between NAFLD and prostate cancer risk (mainly concerning advanced stage tumors). At the same time, Arase et al. [238] noted that the third most commonly found malignancy among NAFLD patients was prostate cancer (12.6%). In another research, Choi et al. [239] suggested that the presence of NAFLD was considered to be protective against prostate cancer biochemical recurrence after radical prostatectomy. According to Allen et al. [197], the highest risk of developing malignancy among NAFLD patients was observed in liver and uterine cancer (incidence rate ratio, 2.3; 95% CI, 1.4–4.1). On the other hand, Huber et al. [236] found that NAFLD was associated with an increased risk of developing skin cancer (irrespectively of sex) and genital cancer in males. Simon et al. [240] mentioned a correlation between NAFLD and a modest increase in kidney/bladder cancer and melanoma. Some authors also showed that high levels of a metabolic risk score composed of five features (blood pressure, BMI, total cholesterol, triglyceride and glucose levels) were associated with renal cell cancer development [241]. Meanwhile, other authors [242] observed an association between NAFLD, obesity and pulmonary adenocarcinoma, particularly among nonsmoking females.

## 4. Conclusions

Overall, NAFLD represents a major cause of liver dysfunction and chronic liver disease globally. It is a silent liver disease, mostly without causing any symptoms. However, as a multisystem disease, NAFLD may lead to severe liver-related and extrahepatic complications, including malignancies. Several researchers have pointed out the possible links between NAFLD and gastrointestinal tract malignancies. Indeed, the association of NAFLD with colorectal adenomas and cancer has been thoroughly investigated during the past decades. Nevertheless, further studies are required to gain a better knowledge and understanding of the mechanisms underlying the association between NAFLD and cancer risk. The presence of NAFLD might act as a prognostic factor for developing extrahepatic cancer. As a result, early NAFLD diagnosis could help prevent the progression of the disease and eventually decrease the incidence and mortality of extrahepatic malignancies.

## Figures and Tables

**Table 1:** Summary of recent studies investigating the association between NAFLD and colorectal neoplasms.

Author, Year	Country	Study Design	Study Population	Diagnosis of NAFLD and Colorectal Neoplasms	Main Findings
Hwang et al., 2010 [174]	South Korea	Cross-sectional study	2917 participants undergoing routine colonoscopy (556 subjects with adenomatous polyps and 2361 subjects without polyps)	US and colonoscopy	NAFLD prevalence (adenomatous polyp group vs. control group): 41.5% vs. 30.2% (*p* < 0.001). NAFLD was associated with an increased risk of developing colorectal adenomatous polyps (OR, 1.28; 95% CI, 1.03–1.60; *p* = 0.029)
Touzin et al., 2011 [175]	USA	Retrospective cohort study	233 patients undergoing screening colonoscopy (94 patients with NAFLD and 139 patients without NAFLD)	Liver biopsy + US, and colonoscopy	Prevalence of colonic adenomas (NAFLD vs. control group): 24.4% vs. 25.1% (*p* = 1.00). Regarding the prevalence of adenomas, no difference was observed between the two groups
Wong et al., 2011 [176]	China	Cross-sectional study	380 community and consecutive patients undergoing screening colonoscopy (199 patients with NAFLD and 181 patients without NAFLD)	Proton-magnetic resonance spectroscopy/ liver biopsy, and colonoscopy.	Prevalence of colorectal adenomas (NAFLD vs. control group): 34.7% vs. 21.5% (*p* = 0.043). Prevalence of advanced colorectal neoplasms (NAFLD vs. control group): 18.6% vs. 5.5% (*p* = 0.002). Among the biopsy-proven NAFLD patients, the prevalence of (a) colorectal adenomas (NASH vs. NAFL group) was 51% vs. 25.6% (*p* = 0.005), and (b) advanced colorectal neoplasms (NASH vs. NAFL group) was 34.7% vs. 14.0% (*p* = 0.011). NASH was associated with colorectal adenomas (adjusted OR, 4.89; 95% CI, 2.04–11.70; *p* < 0.001) and advanced colorectal neoplasms (adjusted OR, 5.34; 95% CI, 1.92–14.84; *p* = 0.001)
Stadlmayr et al., 2011 [177]	Austria	Cross-sectional study	1211 patients undergoing screening colonoscopy (632 patients with NAFLD and 579 patients without NAFLD)	US and colonoscopy	Prevalence of colorectal lesions (NAFLD vs. control group): 34% vs. 21.7% (*p* < 0.001). Among men, (a) the prevalence of rectal adenomas (NAFLD vs. control group) was 11% vs. 3.4% (*p* = 0.004), and (b)CRC prevalence (NAFLD vs. control group) was 1.6% vs. 0.4% (*p* < 0.001). Hepatic steatosis was independently associated with an increased risk of developing colorectal adenomas (adjusted OR, 1.47; 95% CI, 1.079–2.003; *p* = 0.015)
Lee et al., 2012 [178]	South Korea	Retrospective cohort study	5517 females undergoing life insurance health examinations (831 participants with NAFLD and 4686 participants without NAFLD)	US and colonoscopy	NAFLD was independently associated with an increased risk of developing colorectal adenomatous polyps (adjusted RR, 1.94; 95% CI, 1.11–3.40) and CRC (adjusted RR, 3.08; 95% CI, 1.02–9.34)
Min et al., 2012 [179]	South Korea	Retrospective study	227 CRC patients (59 patients with NAFLD and 168 patients without NAFLD)	US and colonoscopy	The presence of NAFLD had no influence on the prognosis of CRC patients. There was no significant difference between CRC patients with and without NAFLD regarding the location and differentiation of tumors, CEA, and the total number of synchronous or advanced colorectal adenomas
Huang et al., 2013 [180]	Taiwan	Retrospective cohort study	1522 participants undergoing two consecutive colonoscopies (216 individuals with colorectal adenomas and 1306 individuals without colorectal adenomas after a negative baseline colonoscopy	US and colonoscopy	NAFLD prevalence (adenoma vs. non-adenoma group): 55.6% vs. 38.8% (*p* < 0.05). NAFLD was an independent risk factor for developing colorectal adenomas after a negative baseline colonoscopy (adjusted OR, 1.45; 95% CI, 1.07–1.98; *p* = 0.016)
Lin et al., 2014 [181]	China	Retrospective and consecutive cohort study	2315 community subjects undergoing routine colonoscopy (263 patients with NAFLD and 2052 patients without NAFLD)	US and colonoscopy	Prevalence of colorectal lesions (NAFLD vs. control group): 90.9% vs. 93.3%. Prevalence of adenomatous polyps (NAFLD vs. control group): 44.5% vs. 55.7%. Prevalence of colorectal malignant neoplasms (NAFLD vs. control group): 29.3% vs. 18% (*p* < 0.05). NAFLD was an independent risk factor for developing colorectal malignant neoplasms (adjusted OR, 1.868; 95% CI, 1.360–2.567; *p* = 0.001)
You et al., 2015 [182]	China	Retrospective cohort study	1314 patients who underwent surgical resection of CRC (127 patients with NAFLD and 1187 patients without NAFLD)	US, and pathological and colonoscopic sample analyses	There was no significant difference in DFS rates between the CRC patient groups with and without NAFLD (*p* = 0.267). After the adjustment for clinicopathologic covariates, the presence of NAFLD was an independent negative risk factor for OS (HR, 0.593; 95% CI, 0.442–0.921; *p* = 0.02), but not for DFS (*p* = 0.270)
Basyigit et al., 2015 [183]	Turkey	Cross-sectional study	127 consecutive patients undergoing colonoscopy (65 patients with NAFLD and 62 patients without NAFLD)	US and colonoscopy	CRC and colorectal adenomas’ prevalence was significantly higher in patients with insulin resistance (*p* = 0.005 and *p* = 0.008, respectively). CRC prevalence was significantly lower in NAFLD patients (*p* = 0.001). The risks of developing colorectal adenomas and cancer were significantly associated with the presence of insulin resistance (OR, 2.338; 95% CI, 1.080–4.993; *p* = 0.003 and OR, 5.023; 95% CI, 1.789–9.789; *p* = 0.001, respectively). CRC risk was increased in patients with insulin resistance but without NAFLD (OR, 5.218; 95% CI, 1.538–7.448; *p* = 0.017)
Bhatt et al., 2015 [184]	USA	Retrospective cohort study	591 patients who completed the liver transplant evaluation process (68 patients with NAFLD and 523 patients without NAFLD)	Liver biopsy/clinical criteria assessment, and colonoscopy	Prevalence of colorectal polyps (NAFLD vs. non-NAFLD group): 59% vs. 40% (*p* = 0.003). NAFLD was a significant predictor of finding a colorectal polyp (adjusted OR, 2.42; 95% CI, 1.42–4.11; *p* = 0.001). Prevalence of adenomatous polyps (NAFLD vs. non-NAFLD group): approximately 32% vs. 21% (*p* = 0.04). NAFLD was a significant predictor of finding colorectal adenomas (adjusted OR, 1.95; 95% CI, 1.09–3.48; *p* = 0.02)
Lee et al., 2016 [185]	South Korea	Cross-sectional study	44,220 participants undergoing colonoscopy and abdominal US as part of a health screening program (14,655 participants with NAFLD and 29,565 participants without NAFLD)	US and colonoscopy	Adjusted ORs for colorectal neoplasms (patients with NAFLD vs. without NAFLD): 1.13; 95% CI, 1.04–1.24 for mild, 1.12; 95% CI, 0.94–1.33 for moderate, and 1.56; 95% CI, 0.98–2.47 for severe NAFLD (*p* for trend = 0.007). Adjusted ORs for non-advanced colorectal neoplasms (patients with NAFLD vs. without NAFLD): 1.12; 95% CI, 1.01–1.23 for mild, 1.10; 95% CI, 0.91–1.33 for moderate, and 1.65; 95% CI, 1.02–2.67 for severe NAFLD (*p* for trend = 0.02). Adjusted ORs for advanced colorectal neoplasms (patients with NAFLD vs. without NAFLD): 1.22; 95% CI, 0.98–1.53 for mild, 1.21; 95% CI, 0.78–1.89 for moderate, and 0.96; 95% CI, 0.23–3.98 for severe NAFLD (*p* for trend = 0.139). Colorectal neoplasm risk increased with worsening fatty liver severity
Pan et al., 2017 [186]	China	Cross-sectional study	1793 participants undergoing colonoscopy and abdominal US as part of health status check-up (573 participants with NAFLD and 1220 participants without NAFLD)	US and colonoscopy	NAFLD was independently associated with an increased risk of developing colorectal neoplasms (adjusted OR, 2.11; 95% CI, 1.352–2.871; *p* = 0.001) and CRC (adjusted OR, 2.164; 95% CI, 1.289–3.217; *p* = 0.005)
Ahn et al., 2017 [187]	South Korea	Cross-sectional study	26,540 participants undergoing colonoscopy and abdominal US as part of a health check-up program (9501 participants with NAFLD and 17,039 participants without NAFLD)	US and colonoscopy	Prevalence of colorectal tumors (NAFLD vs. non-NAFLD group): 38% vs. 28.9% (*p* < 0.001). Prevalence of advanced colorectal neoplasia (NAFLD vs. non-NAFLD group): 2.8% vs. 1.9% (*p* < 0.001). NAFLD was independently associated with an increased risk of developing any colorectal neoplasia (adjusted OR, 1.10; 95% CI, 1.03–1.17; *p* = 0.002), but not advanced colorectal neoplasia (adjusted OR, 1.21; 95% CI, 0.99–1.47; *p* = 0.053)
Chen et al., 2017 [188]	China	Cross-sectional study	3686 individuals undergoing abdominal US and colonoscopy as part of routine health check-up (779 individuals with NAFLD and 2907 individuals without NAFLD)	US and colonoscopy	NAFLD was independently associated with an increased risk of developing colorectal polyps (adjusted OR, 1.26; 95% CI, 1.05–1.51; *p* < 0.05) and colorectal adenomas (adjusted OR, 1.28; 95% CI, 1.01–1.64; *p* < 0.05). Significant association was found between NAFLD and colorectal adenomas in males (adjusted OR, 1.53; 95% CI, 1.18–2.00; *p* < 0.05), but not in females. NAFLD was also associated with multiple colorectal adenomas (OR, 1.82; 95% CI, 1.29–2.55; *p* = 0.001), distal adenomas (OR, 1.63; 95% CI, 1.11–2.39; *p* = 0.013) and bilateral adenomas (OR, 1.89; 95% CI, 1.23–2.91; *p* = 0.004)
Yang et al., 2017 [189]	South Korea	Retrospective cohort study	1023 patients undergoing surveillance colonoscopy after index colonoscopy (unmatched population: 441 patients with NAFLD and 582 patients without NAFLD; propensity score matched population: 441 patients with NAFLD and 441 patients without NAFLD)	US or CT scan, and colonoscopy	Overall colorectal neoplasm occurrence at 3 years after index colonoscopy (NAFLD vs. non-NAFLD group): 9.1% vs. 5% Overall colorectal neoplasm occurrence at 5 years after index colonoscopy (NAFLD vs. non-NAFLD group): 35.2% vs. 25.3% (*p* = 0.01). NAFLD was independently associated with an increased risk of developing colorectal neoplasms (adjusted HR, 1.31; 95% CI, 1.01–1.71; *p* = 0.05) and multiple (≥3) adenomas (adjusted HR, 2.49; 95% CI, 1.20–5.20; *p* = 0.02), but not advanced colorectal neoplasms (adjusted HR, 1.07; 95% CI, 0.51–2.26; *p* = 0.85)
Kim et al., 2017 [190]	South Korea	Cohort study	25,947 subjects undergoing screening colonoscopy as part of a health check-up program (8721 subjects with NAFLD and 17,226 subjects without NAFLD)	US and colonoscopy	NAFLD was significantly associated with CRC in males (adjusted HR, 2.01; 95% CI, 1.10–3.68; *p* = 0.02), but not in females (*p* = 0.41). The severity of NAFLD was not associated with CRC risk
Ze et al., 2018 [191]	South Korea	Retrospective observational study	2976 consecutive subjects undergoing abdominal US and colonoscopy as part of a health check-up program (1512 subjects with NAFLD and 1464 subjects without NAFLD)	US and colonoscopy	Fatty liver index ≥ 30 was associated with an increased risk of developing colorectal adenomas (OR, 1.269; 95% CI, 1.06–1.49; *p* = 0.008)
Chen et al., 2018 [192]	China	Cross-sectional study	764 CRC patients who were primarily treated by surgical resection (316 patients with NAFLD and 448 patients without NAFLD)	US and pathological sample analyses	Significant NAFLD was an independent risk factor for CRC-specific mortality in females. Significant NAFLD and metabolic syndrome has a synergistic effect on promoting mortality among CRC patients
Kim et al., 2019 [193]	South Korea	Cross-sectional study	6332 subjects undergoing abdominal US and 1st-time colonoscopy as part of a health screening program (2395 subjects with NAFLD and 3937 subjects without NAFLD)	US and colonoscopy	Prevalence of colorectal adenomas (NAFLD vs. non-NAFLD group): 33.3% vs. 23.8% (*p* < 0.001). Prevalence of advanced adenomas (NAFLD vs. non-NAFLD group): 5.3% vs. 2.4% (*p* < 0.001). Prevalence of multiple colorectal adenomas (NAFLD vs. non-NAFLD group): 5.8% vs. 3% (*p* < 0.001). NAFLD was independently associated with the risk of developing colorectal adenomas (adjusted OR, 1.15; 95% CI, 1.02–1.30; *p* = 0.027), advanced adenomas (adjusted OR, 1.50; 95% CI, 1.12–2.01; *p* = 0.006), and multiple adenomas (adjusted OR, 1.32; 95% CI, 1.01–1.73; *p* = 0.006)
Hamaguchi et al., 2019 [194]	Japan	Cohort study	15,926 individuals participating in a health check-up program (3211 individuals with NAFLD and 12,715 individuals without NAFLD)	US and colonoscopy	CRC incidence rate: 0.37 per 1000 person years in the non-NAFLD group without obesity; 0.72 in the non-NAFLD group with obesity; 0.41 in the NAFLD group without obesity; 1.49 in the NAFLD group with obesity. NAFLD with obesity was independently associated with an increased CRC risk (adjusted HR, 2.96; 95% CI, 1.44–6.09; *p* = 0.003)
Li et al., 2019 [195]	China	Retrospective cohort study	1089 subjects undergoing colonoscopy (502 subjects with NAFLD and 587 subjects without NAFLD)	US + CAP score using FibroScan probes, and colonoscopy	NAFLD was independently associated with an increased risk of developing colorectal adenomas (OR, 1.425; 95% CI, 1.112–2.042; *p* = 0.018). NAFLD was associated with an increased adenoma risk in males (OR, 1.473; 95% CI, 1.003–2.162; *p* = 0.048), but not in females (OR, 1.316; 95% CI, 0.817–2.12; *p* = 0.259). NAFLD and metabolic syndrome were significantly associated with a high risk of developing adenomas
Cho et al., 2019 [196]	South Korea	Prospective cohort study	476 patients undergoing screening colonoscopy (379 patients with NAFLD and 97 patients without NAFLD)	Liver biopsy and colonoscopy	NAFL was independently associated with an increased risk of developing adenomatous polyps (adjusted OR, 2.76; 95% CI, 1.51–5.06; *p* = 0.001). NASH was independently associated with an increased risk of developing colorectal adenomatous polyps (adjusted OR, 2.08; 95% CI, 1.12–3.86; *p* = 0.02) and advanced colorectal neoplasms (adjusted OR, 2.81; 95% CI, 1.01–7.87; *p* = 0.049)
Allen et al., 2019 [197]	USA	Cohort study	19,163 subjects (4722 subjects with NAFLD and 14,441 age- and sex-matched referent individuals)	NAFLD and cancer was defined utilizing a code-based algorithm (using the NAFLD-specific HICDA, ICD-9-CM and ICD-10-CM codes)	NAFLD was associated with an increased colon cancer risk (IRR, 1.8; 95% CI, 1.1–2.8)
Lee et al., 2020 [198]	South Korea	Retrospective cohort study	8,120,674 subjects who received healthcare checkups (936,159 adults with NAFLD and 7,184,515 adults without NAFLD)	FLI, and endoscopy + ICD-10 codes	NAFLD (FLI ≥ 60) was significantly associated with the risk of developing colon cancer (HR, 1.23; 95% CI, 1.19–1.26) and an increased risk of all-cause mortality in CRC patients (HR, 1.16; 95% CI, 1.10–1.22)
Blackett et al., 2020 [199]	USA	Cross-sectional study	369 patients who underwent liver biopsy and screening or surveillance colonoscopy (123 subjects with NAFLD and 246 matched controls without NAFLD)	Liver biopsy and colonoscopy	Prevalence of colorectal adenomas (NAFLD vs. control group): 40.7% vs. 28.1% (OR, 1.87; 95% CI, 1.15–3.03; *p* = 0.01). NAFLD was independently associated with an increased risk of detecting colorectal adenomas (adjusted OR, 1.74; 95% CI, 1.05–2.88; *p* = 0.032), but not advanced neoplastic lesions (adjusted OR, 2.2; 95% CI, 0.93–5.18; *p* = 0.07). The risk of developing colorectal adenomas was not associated with the severity (steatohepatitis vs. no steatohepatitis) of NAFLD (adjusted OR, 2.47; 95% CI, 0.67–9.1; *p* = 0.17)
Lesmana et al., 2020 [200]	Indonesia	Retrospective database study	138 subjects undergoing elective colonoscopy (68 subjects with NAFLD and 70 subjects without NAFLD)	US and colonoscopy	Prevalence of colon polyps (NAFLD vs. control group): 44.1% vs. 27.1% (*p* = 0.037). NAFLD was associated with an increased risk of developing any colon polyp
Yu et al., 2020 [201]	China	Cross-sectional study	1538 patients with colorectal polyps undergoing abdominal US (550 patients with NAFLD and 988 patients without NAFLD)	US and colonoscopy	No significant difference regarding the location and morphology of colorectal polyps between the NAFLD and control groups (*p* > 0.05). NAFLD was significantly associated with colorectal polyps, especially, in patients with multiple polyps, those with a large size and with villous features (*p* < 0.05)
Zhang et al., 2021 [202]	China	Retrospective cohort study	8351 NAFLD patients (5308 patients with prior colonoscopy and 3043 patients without prior colonoscopy)	- CRC was identified based on ICD-9-CM diagnosis codes or procedure codes for CRC treatment	Compared to the general population, NAFLD patients who did not undergo colonoscopy had higher incidence rate of CRC (SIR, 2.20; 95% CI, 1.64–2.88; *p* < 0.001). NAFLD patients who underwent colonoscopy had lower incidence rate of CRC (SIR, 0.54; 95% CI, 0.37–0.75; *p* < 0.001). After adjustment for demographic and metabolic factors, NAFLD patients with a high fibrosis-4 score (>2.67) had higher risk of developing CRC
Fukunaga et al., 2021 [203]	Japan	Cross-sectional study	124 consecutive health check-up examinees undergoing colonoscopy (58 examinees with NAFLD and 66 examinees without NAFLD; 63 examinees with MAFLD and 61 examinees without MAFLD)	US and colonoscopy	MAFLD was independently associated with colorectal adenomas (OR, 3.191; 95% CI, 1.494–7.070; *p* = 0.003). Non-obese MAFLD was also associated with colorectal adenomas (OR, 3.351; 95% CI, 1.589–7.262; *p* ≤ 0.001)
Kim et al., 2021 [204]	South Korea	Cohort study	6182 subjects undergoing abdominal US, endoscopic removal of ≥1 adenomas at the index colonoscopy and a follow-up surveillance colonoscopy (2642 subjects with NAFLD and 3540 subjects without NAFLD)	US and colonoscopy	NAFLD was independently associated with an increased risk of developing metachronous overall colorectal neoplasia in both males (adjusted HR, 1.17; 95% CI, 1.06–1.29) and females (adjusted HR, 1.63; 95% CI, 1.27–2.07). NAFLD was also independently associated with an increased risk of developing metachronous advanced colorectal neoplasia in females (adjusted HR, 2.61; 95% CI, 1.27–5.37)
Seo et al., 2021 [205]	South Korea	Retrospective cohort study	A total of 3441 subjects participating in a health check-up program (1127 subjects with MAFLD and 2314 without MAFLD). 3044 subjects were included in the NAFLD analysis (1143 subjects with NAFLD and 1901 subjects without NAFLD)	US and colonoscopy	NAFLD and MAFLD were significantly associated with an increased risk of developing colorectal adenomas in females (adjusted OR, 1.43; 95% CI, 1.01–2.03; *p* = 0.046 and OR, 1.55; 95% CI, 1.09–2.20; *p* = 0.015, respectively). NAFLD and MAFLD with an advanced fibrosis index score were also associated with an increased risk of developing adenomas (OR, 1.38; 95% CI, 1.04–1.83; *p* = 0.027, and OR, 1.45; 95% CI, 1.13–1.96; *p* = 0.004, respectively)
Lee et al., 2022 [206]	South Korea	Cohort study	8,933,017 participants undergoing routine National Health Insurance Service health examinations (2,517,330 participants with NAFLD and 6,415,687 participants without NAFLD; 3,337,122 participants with MAFLD and 5,595,895 participants without MAFLD)	FLI, and ICD-10 diagnosis codes	The presence of fatty liver disease was significantly associated with an increased CRC risk. The CRC risk was higher in MAFLD patients with liver fibrosis

NAFLD: non-alcoholic fatty liver disease; US: ultrasonography; OR: odds ratio; CI: confidence interval; NASH: non-alcoholic steatohepatitis; CRC: colorectal cancer; RR: relative risk; CEA: carcinoembryonic antigen; DFS: disease-free survival; OS: overall survival; HR: hazard ratio; CT: computed tomography; CAP: controlled attenuation parameter; HICDA: Hospital International Classification of Diseases Adapted; ICD: International Classification of Diseases; CM: clinical modification; IRR: incidence rate ratio; FLI: fatty liver index; SIR: standardized incidence ratio; MAFLD: metabolic dysfunction-associated fatty liver disease.
